# Breaking the cycle: long-term socio economic determinants of child labour in SAARC countries

**DOI:** 10.1186/s12889-025-25399-w

**Published:** 2025-11-19

**Authors:** Tharaka Magammana, Himashi Muthugala, Amanda Bandara, Ayodhya Perera, Ruwan Jayathilaka

**Affiliations:** 1 SLIIT Business School, Sri Lanka Institute of Information Technology, New Kandy Road, Malabe, Sri Lanka; 2 Head - Department of Information Management, SLIIT Business School, Sri Lanka Institute of Information Technology, New Kandy Road, Malabe, Sri Lanka

**Keywords:** Child labour, Education, Health, Economic growth, FDI, Unemployment, Urbanisation, SAARC

## Abstract

**Background:**

Child labour remains a critical issue in SAARC countries, driven by various socio-economic factors. While previous studies have explored individual determinants, limited research has been conducted on their collective long-term impact. Understanding how structural and economic conditions shape child labour trends is essential for designing effective policy interventions.

**Methods:**

This study engages panel cointegration techniques to examine the long-term relationship between child labour and key socio-economic drivers in SAARC countries. It assesses the impact of education, access to healthcare, economic conditions, labour market dynamics, foreign investment, and urbanisation on the prevalence of child labour.

**Results:**

The findings confirm a stable, long-term relationship between child labour and these determinants in each SAARC country. Improvements in education and health significantly reduce child labour. However, economic growth and urbanisation have complex, country-specific effects. Higher unemployment and increased FDI may also influence child labour, emphasising the need for targeted policy responses.

**Conclusions:**

The study highlights the significance of ongoing investments in education and healthcare. Labour market reforms are crucial to mitigate the impact of unemployment, while inclusive economic policies ensure that growth benefits vulnerable populations. Targeted strategies for FDI and urbanisation are necessary to prevent unintended consequences on child labour. Combating child labour in SAARC countries requires a multi-sectoral approach. Regional collaboration is crucial for sharing best practices, developing unified strategies, and enhancing cross-border initiatives. Holistic policies integrating education, health, and economic planning are key to reducing child labour.

**Supplementary Information:**

The online version contains supplementary material available at 10.1186/s12889-025-25399-w.

## Introduction

### Background of the study

Child labour remains one of the most persistent global development challenges, affecting an estimated 138 million children between the ages of 5 and 17 worldwide, with around 54 million engaged in hazardous work as of 2024 [1]. Despite international commitments such as SDG 8.7, which calls for the elimination of child labour by 2030, progress has been uneven, and current reduction rates remain insufficient to meet this target [2].

The consequences of child labour extend beyond immediate hardship. Research consistently links child labour to restricted physical growth, cognitive impairment, and long-term psychological distress [3–5]. At the societal level, child labour reinforces intergenerational poverty and inequality, reducing human capital accumulation and hindering economic development [6, 7]. These multidimensional impacts underscore the need to investigate not only the economic drivers of child labour but also the broader structural and social conditions that perpetuate it.

### Focus on SAARC countries

South Asia accounts for some of the highest rates of child labour globally, with over 41 million children aged 5–14 engaged in work, many of them in hazardous conditions and excluded from education [1, 8]. Within the SAARC region, countries such as Nepal and Afghanistan record particularly severe prevalence rates [9]. The persistence of child labour in these nations is driven by entrenched poverty, limited access to quality education, weak enforcement of labour regulations, and systemic social inequalities [10, 11].

In the South Asian context, the region’s socio-economic diversity further shapes child labour dynamics. Several factors contribute to this issue, including low-quality education systems, rapid urbanisation, low economic growth rates, fluctuating cash inflows, and uneven health outcomes, which together create varied patterns of vulnerability across SAARC countries [12–14]. For example, while some economies are experiencing structural shifts that reduce reliance on child labour, others continue to depend heavily on informal and agricultural labour markets where children are excessively employed. These contrasting realities make the SAARC region a critical case for studying the long-term determinants of child labour.

By focusing specifically on SAARC countries, this study addresses an important gap in the literature. Much of the existing research has concentrated on single countries or isolated factors, overlooking the broader regional context and the interplay of multiple socio-economic determinants. A regional analysis not only captures shared structural challenges but also highlights cross-country variations that are essential for developing effective, locally adapted policy responses.

### Research gap and rationale

While child labour has been extensively studied, research on the SAARC region remains limited and fragmented. Existing work often focuses on single determinants such as poverty, education, or health [15, 16], or on individual countries, overlooking the interplay of multiple socio-economic drivers across the region. Methodologically, most prior studies have relied on short-term or linear models, failing to capture the persistent and long-run dynamics of child labour [17].

This study addresses these gaps by systematically examining the long-term relationships between child labour and six key determinants: education, health, economic growth, unemployment, urbanisation, and foreign direct investment (FDI) across SAARC countries using panel cointegration techniques. By integrating social and economic dimensions into a unified regional framework, the study enhances theoretical and methodological understanding and provides evidence-based insights for policy interventions in the SAARC context.

### Research objectives and research questions

The primary objective of this study is to examine the long-term relationship between child labour and its key socio-economic determinants in SAARC countries. Using a panel cointegration framework, the study seeks to understand how social and economic factors collectively shape child labour dynamics in the region over time.

#### Social factors

This study investigates the influence of education and health on child labour trends. It aims to determine how educational attainment affects the prevalence of child labour and to assess the role of health outcomes in shaping child labour patterns across SAARC countries in the long term.

#### Economic factors

The study further explores the impact of economic growth, unemployment, urbanisation, and FDI on child labour. It examines how sustained economic growth and labour market conditions influence child participation in work, while also analysing the effects of urbanisation on child labour patterns. Additionally, the study considers how FDI may affect socio-economic conditions that indirectly shape child labour in these countries.

By addressing these social and economic dimensions, the study aims to provide a nuanced understanding of the determinants of child labour, offering insights that can inform policy formulation and development strategies within the SAARC region.

This study makes three key contributions. First, it advances theory by integrating six socio-economic determinants including education, health, economic growth, unemployment, urbanisation, and FDI into a comprehensive regional framework, offering a more holistic understanding of child labour in SAARC countries. Second, it contributes methodologically by applying a panel cointegration approach, allowing identification of both short-term and long-term relationships often overlooked in prior studies. Third, it provides practical value through evidence-based insights that inform policies aimed at addressing the structural causes of child labour, supporting SAARC governments and international organisations in developing targeted interventions.

## Literature review

### Theoretical framework

#### Human capital theory and child labour

Human Capital Theory is one of the most common ways to explain the link between education, labour and development. The idea, first explained by [18], is that education and skills are like “capital,” just like land or machines, because they help people become more productive and earn more in the future. In poor countries, including many in South Asia, families often face a choice between sending children to school or putting them to work. From this perspective, investing in schooling reduces child labour in the long run because it raises the returns to education [19, 20], and gives children better chances of decent work as adults.

A key part of this theory is the “opportunity cost” of schooling. For poor households, the income a child could earn by working may seem more valuable than the uncertain benefits of education later. This is why many children in India, Bangladesh and Nepal still go to work, even when wages are very low [21]. In the short run, child labour helps families survive, but in the long run it limits the child’s human capital and traps families in poverty across generations [22]. At the same time, studies show that when education is affordable and families believe it leads to better jobs, they are much more likely to keep children in school [17, 20]. Evidence also shows that each extra year of schooling increases income by around 8–10% on average, which highlights why investing in education is such an important strategy against child labour [19], especially in SAARC countries where the rates are alarmingly increasing. By using this theory, the present study shows how social variables such as education and health affect child labour.

#### Poverty trap theory and child labour

The Poverty Trap Theory explains how poor families remain stuck in a cycle where today’s poverty causes tomorrow’s poverty [23, 24]. Limited resources prevent investment in education, healthcare, or productive assets, so children grow up with fewer opportunities and remain poor as adults. Child labour is both a consequence and a cause of this cycle. HC Fors [25]HC Fors [25] notes that while child labour may help families survive short-term shocks, it reinforces long-term poverty. Parents often rely on children’s income when adult wages are insufficient. This is a concept by Basu and PH Van [22]Basu and PH Van [22] call the “luxury axiom.” In South Asia, households with little savings or access to credit frequently send children to work during unemployment, illness, or crop failure [26]. This perpetuates the cycle: children miss school, acquire fewer skills, and earn less as adults, often depending on their own children’s labour.

Empirical evidence supports this link [6]. show that cash transfers reduce child labour, while [27] highlight its long-term negative impact on human capital. In SAARC countries, poverty and weak social protection drive child labour in Bangladesh, Nepal, Pakistan, and Afghanistan, while rural and migrant families in India and Sri Lanka also remain vulnerable [28, 29]. Policy solutions require addressing poverty’s root causes, not just banning child labour. Measures like cash transfers, free school meals, better education and health systems, and stable adult employment can help break the intergenerational cycle [6]. The selection of variables such as unemployment, economic growth, and urbanisation is closely linked to the poverty trap theory, as these factors directly affect household income and job security. By examining their impact, the study explores how economic conditions influence families’ reliance on child labour and the persistence of intergenerational poverty.

## Social determinants of child labour

### Health and child labour

The relationship between child labour and health is a persistent concern across SAARC countries, as children engaged in hazardous and labour-intensive activities face substantial physical and psychological risks. Empirical studies consistently show that child labourers experience respiratory illnesses, musculoskeletal disorders, malnutrition, and frequent injuries due to insecure workplaces [30–32]. In Pakistan, children employed in leather and surgical industries are routinely exposed to toxic chemicals, leading to chronic health problems [15], while in Bangladesh and India, child labourers in construction, manufacturing, and agriculture face high risks of accidents and long-term evolving impairments [33, 34]. Evidence from Afghanistan indicates that child labourers, particularly among refugee populations, often suffer chronic fatigue and psychological distress (Singh, 1983). In Nepal, workplace injuries remain widespread [35, 36], while nearly 28% of working children in Sri Lanka report occupation-related health conditions [37]. However, these studies often lack cross-country context, as most focus on single-country data.

While the physical health risks of child labour are well documented, research on its long-term mental health consequences is rare. Gender disparities further worsen vulnerabilities, as girls are disproportionately exposed to reproductive health risks and exploitation [38]. Comparative research across SAARC countries is also limited, making it problematic to evaluate the relative effectiveness of health interventions. Some country-level initiatives, such as Bangladesh’s community health programmes and Sri Lanka’s family support schemes, have enhanced healthcare access for child labour [39, 40], while India has introduced workplace safety regulations with mixed success. However, in conflict-affected and economically unstable contexts such as Afghanistan and Pakistan, enforcement remains weak.

Taken together, the literature underscores that although health impacts of child labour are widely recognised, gaps remain in understanding the psychological consequences, gendered risks, and cross-country variations. Moreover, few studies examine the long-term interplay between child health and wider socio-economic conditions such as poverty, education, and labour market structures. This highlights the need for regional, comparative analyses that capture these dynamics within a unified framework.

#### Education and child labour

Education is widely recognised as a main mechanism for reducing child labour, yet across SAARC countries its protective effect is uneven due to deep-rooted socio-economic barriers. In Pakistan, financial hardship and insufficient school infrastructure drive high dropout rates, pushing children into labour despite the long-term benefits of education [41, 42]. Similar limitations exist in India, where poverty, caste-based inequalities, and fragile governance of child labour regulations continue to undermine educational access [16, 43]. In Afghanistan, conflict and economic collapse have left millions of children without formal schooling [44], worsening cognitive and socio-emotional deprivation.

Bangladesh provides an important contrast: school stipends, free meal programmes, and vocational training schemes have reduced child labour by improving educational incentives [28]. Nevertheless, these policies have produced unintended outcomes, such as rising dropout rates despite a decline in labour participation (Emran, 2024). Similarly, in Nepal, community-based interventions and legal awareness campaigns have promoted schooling as an alternative to child labour, but rural households frequently persist dependent on child income [45, 46]. In Sri Lanka, vocational training and support for vulnerable families have helped prevent school dropouts, though little empirical research has directly assessed their impact on child labour [37, 47]. Taken together, these findings reveal that while education consistently decreases child labour, its effectiveness depends heavily on the broader socio-economic and institutional context. Interventions such as stipends and community awareness can be effective, but their success is undermined by persistent poverty, gender inequalities, and poor regulatory compliance of labour and education policies. The literature therefore points to the need for integrated approaches that address not only schooling access but also the structural constraints that limit its effectiveness.

### Economic determinants of child labour

#### Economic growth and child labour

The relationship between economic growth and child labour in SAARC countries is multifaceted and non-linear. On one hand, growth can raise household incomes and reduce reliance on child labour; on the other hand, early stages of industrial expansion often create new demand for low-skilled, cheap labour, which may include children [48, 49]. This duality is reflected in country-level evidence. In India, while sustained growth has lifted many families out of poverty, weak enforcement of labour regulations has allowed child employment to persist in hazardous industries such as brick kilns and fireworks [50]. In Afghanistan, widespread poverty and economic uncertainty have left growth benefits largely inaccessible to vulnerable households [44], compelling children into hazardous work to support family survival.

Other studies suggest that growth may diminish child labour when it is inclusive and accompanied by structural reforms. For instance, in Pakistan, economic growth has improved adult wages in certain sectors, lowering the requirement of child labour for some households [51]. Yet, growth in informal or unregulated sectors often continues to rely on children, reinforcing inequalities and limiting opportunities for education [52, 53]. Marginalised rural communities across the region remain particularly vulnerable, as growth is irregularly distributed and fails to dismantle structural barriers that drive child labour.

Overall, the literature shows that economic growth alone is not a necessary condition for reducing child labour. Its impact depends on how equitably the benefits of growth are distributed and whether effective labour protections, education systems, and social policies are in place. This underlines the importance of examining growth in conjunction with other socio-economic factors, rather than in isolation, to better understand its long-term consequences for child labour in SAARC countries.

#### FDI and child labour

FDI plays a vital role in shaping labour markets in developing economies, including those in the SAARC region. Its impact on child labour, however, is highly context-dependent and differs across sectors and countries. In many cases, FDI increases adult wages and improves household welfare, thereby reducing the necessity for children to contribute to family income [54–56]. Mechanisation associated with FDI can also lower the demand for low-skilled labour, displacing child labour from certain industries.

At the same time, evidence suggests that FDI can exacerbate child labour. when it flows into labour-intensive, weakly regulated segments. In Pakistan, for example, globalisation and trade liberalisation have had mixed impacts: while international pressure has pushed some industries to expand labour standards, limited institutional capacity has allowed others to continue relying on child labour [57]. Similarly, sectoral differences are critical: manufacturing investment tends to reduce child labour, while agricultural FDI, particularly in informal or subsistence settings, may increase reliance on children for cheap labour [58]. Wage inequality also remains a concern, as FDI often benefits skilled workers disproportionately [24, 59], leaving poorer households vulnerable to child labour dependence.

Overall, the literature shows that FDI’s impact on child labour is neither uniformly positive nor negative. Its effects depend on the sectoral composition of investment, the strength of regulatory frameworks, and the extent to which gains are equitably distributed. This mixed evidence highlights the need for comparative, region-wide analysis of how FDI interacts with broader socio-economic conditions in SAARC countries, particularly in relation to education, urbanisation, and labour market policies.

#### Urbanisation and child labour

Urbanisation has emerged as a defining feature of socio-economic change in SAARC countries, offering both opportunities and risks for child labour. On one hand, migration to urban centres can improve access to education, healthcare, and social services, reducing children’s engagement in labour. Studies from Nepal and India show that proximity to schools in urban areas often increases enrolment and lowers participation in agricultural labour [60, 61]. Similarly, in Bangladesh, school infrastructure and social safety-net programmes in urban areas have reduced the occurrence of child labour for some communities [62]. On the other hand, rapid and unregulated urbanisation often exposes children to exploitative forms of work in informal sectors. Evidence from Pakistan highlights that migrant and refugee children, specifically Afghan refugees, are pushed into hazardous urban jobs under conditions of poor regulation and limited state protection [63]. In India, rural-to-urban migration has driven children into construction, domestic labour, and restaurant work, often forcing them to drop out of school [64]. In Bangladesh, economic migration to Dhaka has created new urban child labour markets [31], reflecting persistent poverty and lack of affordable schooling.

Taken together, these findings illustrate that urbanisation has contradictory effects: while it can facilitate better access to schooling and social services, it simultaneously expands informal and unregulated employment opportunities for children. The literature shows limited cross-country comparison of these dynamics, and few studies assess how urbanisation interacts with other socio-economic factors, such as unemployment and FDI. This underlines the need for a regional perspective that captures the diverse ways in which urbanisation shapes child labour across SAARC nations.

#### Unemployment and child labour

Unemployment is one of the most consistent drivers of child labour in SAARC countries, as families often depend on children’s earnings when adult job openings are scarce. Cross-country evidence shows that when adult unemployment rises, households are more likely to rely on child labour to compensate for income losses [21, 65]. This relationship is particularly pronounced in contexts of weak social protection and limited access to credit.

In India, high unemployment combined with caste-based inequalities, small landholdings, and inadequate school infrastructure has historically entrenched child labour [66, 67]. The COVID-19 pandemic further worsened urban unemployment, pushing many children into informal and hazardous jobs as families sought economic stability [68]. In Pakistan, unemployment and poverty operate together with weak governance and enforcement, reinforcing both economic hardship and educational exclusion [41, 69]. In Afghanistan, conflict-driven joblessness leaves households with few alternatives, forcing children into exploitative work environments.

Although the adverse link between unemployment and child labour is well documented, studies also show that policy interventions can mitigate this effect. For example, improvements in adult education, tax incentives, and childcare policies have been shown to reduce household reliance on children’s earnings in other developing contexts [70, 71]. Nonetheless, such evidence is limited for SAARC countries, where interventions remain fragmented and irregular.

Overall, the literature underscores that unemployment is a structural determinant of child labour, but the strength of this relationship differs across SAARC depending on institutional capacity, social protection systems, and labour market dynamics. Few comparative studies assess these cross-country variations, pointing to the need for integrated regional analyses that link unemployment with other socio-economic drivers of child labour.

### Research gap and contribution

Although the relationship between child labour and socio-economic conditions has been generally studied in the past, the existing literature focused on SAARC countries remains uneven and often limited in scope. Most of these studies concentrate on single factors such as poverty, education, or health or studies that focus on individual countries, without providing a comprehensive cross-country analysis [15, 16, 72]. As a result, the broader structural interplay of factors such as unemployment, urbanisation, and FDI remains underexplored in the South Asian context.

Another gap within the past studies lies in methodology. Much of the prior research relies on descriptive statistics or linear short-term models, which fail to capture the persistence of child labour and the long-run changing aspects that shape its patterns. This restricts understanding of how multiple socio-economic determinants interact over time to influence child labour outcomes. Furthermore, while country-level evidence is rich, cross-country comparative studies in the SAARC region are scarce, leaving policymakers with little guidance on regional commonalities and differences.

This study addresses these gaps by integrating six key socio-economic determinants education, health, economic growth, unemployment, urbanisation, and FDI into a comprehensive regional framework. By applying a panel cointegration approach, it examines both short-run and long-term equilibrium relationships, offering a methodological advancement over previous studies. In doing so, the study not only extends the theoretical and empirical understanding of child labour but also delivers policy-relevant insights tailored to the shared and country-specific realities of SAARC nations.

## Data and methodology

### Data sources and selection criteria

This study utilises secondary panel data from 2010 to 2022 to investigate the socio-economic determinants of child labour in SAARC countries, excluding the Maldives due to a lack of available data. Data limitations primarily constrain the timeframe, particularly the unavailability of child labour statistics for Afghanistan before 2010. The authors forecasted selected child labour data to address gaps in specific years for some countries. The analysis incorporates six key socio-economic variables, education, health, economic growth, unemployment, urbanisation, and FDI, to comprehensively understand their long-term relationship with child labour. Table 1 presents the data sources and variable descriptions, while the complete dataset is available in Appendix 1.

**Table 1:** Data sources and variables

Variable	Measure	Data Source
Child Labour	Percentage of 5–14 aged population children	U.S. DEPARTMENT OF LABOUR
Health	Health Index (Life expectancy at birth)	Global Data Lab – Substantial Human Development Indexes
Education	Education Index (Mean years of schooling of adults aged 25+, Expected years of schooling of children aged 6)	Global Data Lab – Substantial Human Development Indexes
Economic Growth	Income Index (Gross National Income per capita -PPP, 2011 US$)	Global Data Lab – Substantial Human Development Indexes
Unemployment	Total (% of labour force)	World Bank Indicators
FDI	% of GDP	World Bank Indicators
Urbanisation	% of Population	World Bank Indicators

Source: Compiled by authors

### Variable selection and measurements

In this study, child labour is defined as work that deprives children of their childhood and education, thereby hindering their physical and mental development [9]. It is measured as the percentage of children aged 5–14 engaged in economic activities, encompassing both market and non-market work. The independent variables were selected based on theoretical and empirical evidence highlighting their influence on child labour in South Asia:

Education is proxied by average years of schooling among individuals aged 25+, following the UNDP’s HDI methodology. Education raises the opportunity cost of child labour, thereby reducing its prevalence [21, 22]. South Asian studies similarly show that higher schooling levels are associated with lower child labour [73, 74]. Health, measured by life expectancy at birth, affects children’s capacity to work and attend school. Children in poor health are more vulnerable to entering the labour force [17, 75]. Economic growth (GDP per capita, PPP) influences household income and overall labour demand, thereby generally reducing the incidence of child labour [76, 77].

Unemployment (% of the labour force without work but actively seeking employment) can increase child labour, as children may need to supplement household income when adults are unemployed [21, 78]. FDI (% of GDP) affects labour markets and household income, with mixed evidence on its impact on child labour in developing countries [79, 80]. Urbanisation (% of population in urban areas) reflects structural transformation; greater access to education and services can reduce child labour, however, persistent urban poverty may offset these potential benefits [73]. Collectively, these variables capture the multidimensional economic, social, and structural determinants of child labour in the SAARC region.

### Ethical considerations

This study adheres to ethical considerations by utilising secondary data from the DOL, which is collected under strict supervision and ethical guidelines to protect the rights and confidentiality of participants [81]. By relying on verified datasets, the research negates the need for direct interaction with vulnerable populations, thereby minimising potential risks. Additionally, it upholds ethical standards by transparently acknowledging data sources and complying with regulations regarding the handling of sensitive information.

### Methodological framework

**Fig. 1:**
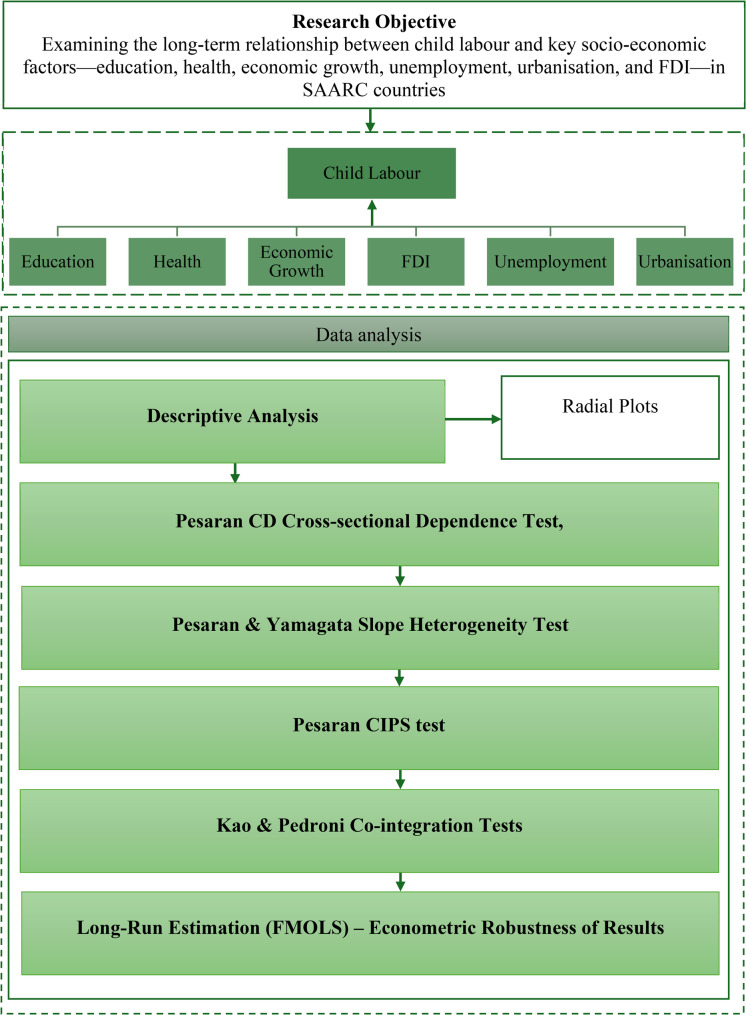
Research Design

This study adopted a structured approach to data processing and analysis, beginning with descriptive statistics and graphical methods to illustrate socio-economic disparities across SAARC countries using STATA software. As shown in Fig. 1, the econometric strategy followed a sequence of diagnostic and inferential steps to ensure methodological robustness.

As a first step, preliminary tests were conducted to assess the properties of the panel dataset. The results of these analysis are presented in Appendix 3. Pesaran’s test of cross-sectional dependence indicated weak inter-country dependence, showing the results were not affected by any interdependence between the variables [82]. The authors next commenced the Pesaran–Yamagata slope heterogeneity test, which confirmed heterogeneity in coefficients across panels. Given these results, the reliance on a single first-generation unit root test would have been inappropriate, as model heterogeneity could affect stationarity [83]. To address this issue, unit root behaviour was assessed through Pesaran CIPS, a second-generation unit root test, which showed that most variables were non-stationary at levels. These findings justified the application of cointegration analysis to examine the long-term equilibrium relationship between child labour and its socio-economic determinants [84]. To assess potential multicollinearity, the study applied a Variance Inflation Factor (VIF). As shown in Appendix 3D, the VIF results indicate that multicollinearity is not a serious concern in our model, as all individual VIF values are below the commonly used threshold of 10, with a mean VIF of 4.26 [85, 86]. This confirms that the estimated long-run relationships among the socio-economic determinants and child labour are unlikely to be biased by multicollinearity.

For the cointegration analysis, authors first employed the Kao test to examine the long-term relationships in the panel data. The Kao test is particularly suitable for panel datasets, as it accounts for country-specific characteristics while estimating long-term relationships [87]. Each country in the panel may have unique characteristics that influence the relationships between the variables, and the Kao test ensures that these differences are correctly accounted for [88].

In order to further addresses the heterogeneity observed across countries, the Pedroni test was also applied. The Pedroni test allows for individual heterogeneity in both intercepts and trend coefficients, enhancing flexibility compared to the Kao test [89]. Using both tests strengthens the robustness of the findings by confirming long-term equilibrium relationships under different assumptions. Recent literature supports the application of cointegration analysis in studying the long-term determinants of child labour, particularly where relationships with socio-economic indicators such as education and income are of interest [90, 91]. Despite concerns regarding inconsistent and repeated data, prior work has shown that cointegration techniques can yield valid results under data irregularities [92].

Finally, to estimate the magnitude of long-run effects while adding the analysis an econometric robustness, the study employed Fully Modified Ordinary Least Squares (FMOLS). This corrects for serial correlation and endogeneity in cointegrated systems [93]. FMOLS provides consistent estimates of long-run elasticities, making it particularly suitable for evaluating how structural socio-economic factors shape child labour dynamics across countries [94].

Building on this, the study also applies Dynamic OLS (DOLS) as a robustness check [95]. Using both FMOLS and DOLS ensures that the long-run coefficients are not sensitive to estimation technique.

To formalise the empirical framework, the following long-run regression equation is specified:

Linear specification:

1 $$\begin{aligned}\:{\text{C}\text{L}}_{\text{i}\text{t}}=&\:{\beta\:}_{0}+\:{{\upbeta\:}}_{1}{\text{E}\text{D}\text{U}}_{\text{i}\text{t}\:}+{{\upbeta\:}}_{2}{\text{H}\text{E}\text{A}}_{\text{i}\text{t}\:}\\&+\:{{\upbeta\:}}_{3}{\text{E}\text{G}}_{\text{i}\text{t}\:}+{{\upbeta\:}}_{4}{\text{U}\text{N}\text{E}}_{\text{i}\text{t}\:}\\&+\:{{\upbeta\:}}_{5}{UB}_{\text{i}\text{t}\:}+\:{{\upbeta\:}}_{6}{\text{F}\text{D}\text{I}}_{\text{t}\:}+\:{\in\:}_{it}\end{aligned}$$

where $\:{CL}_{t}$​ denotes the incidence of child labour in country $\:\left(i\right)$ at time ($\:t)$ the explanatory variables represent socio-economic determinants; $\:{\beta\:}_{0}$ captures country-specific effects; and $\:{\in\:}_{it}$​ is the error term.

In addition to the linear specification, the log–log form was also estimated by the authors for three main reasons. First, some of the coefficients in the linear specification, particularly in education and health, were unusually large in magnitude which raised concerns of scaling distortions. Thus, transforming the variables into natural logarithms alleviates this issue by standardising measurement units [96]. Second, the log–log model enables direct interpretations of coefficients as elasticities, which is more policy-relevant, since it shows the percentage change in child labour associated with a 1% change in each determinant. Third, log transformation helps reduce skewness in variables such as FDI and economic growth and may lessen heteroskedasticity, thereby improving model fit and robustness [97]. For these reasons, both linear and log–log specifications are reported, with the latter included primarily as a robustness exercise and presented in the appendix.

2 $$\begin{aligned}\:\text{ln}\left({\text{C}\text{L}}_{\text{i}\text{t}}\right)=&\:{\beta\:}_{0}+\:{{\upbeta\:}}_{1}ln{(\text{E}\text{D}\text{U}}_{\text{i}\text{t}\:})+{{\upbeta\:}}_{2}\text{ln}\left({\text{H}\text{E}\text{A}}_{\text{i}\text{t}\:}\right)\\&+{{\upbeta\:}}_{3}\text{ln}\left({\text{E}\text{G}}_{\text{i}\text{t}\:}\right)+{{\upbeta\:}}_{4}\text{ln}\left({\text{U}\text{N}\text{E}}_{\text{i}\text{t}\:}\right)\\&+{{\upbeta\:}}_{5}\text{ln}\left({UB}_{\text{i}\text{t}\:}\right)+{{\upbeta\:}}_{6}\text{ln}\left({\text{F}\text{D}\text{I}}_{\text{i}\text{t}\:}\right)+\:{\in\:}_{it}\end{aligned}$$

Additionally, recent empirical literature has contributed valuable insights into the determinants of child labour, highlighting the significance of socio-economic variables such as household income, unemployment, globalisation, and family conditions [7, 78, 80]. These studies, while using panel data, collectively reinforce the importance of structural economic factors in shaping child labour outcomes. This supports the present study’s variable selection and theoretical focus.

## Results

This study examines the long-term relationship between child labour and key socio-economic factors, including education, health, economic growth, unemployment, FDI, and urbanisation, across SAARC countries. Using the Kao cointegration test, the findings reveal that child labour in all analysed countries shares a stable long-term relationship with these variables, highlighting the persistent influence of economic and social conditions on child labour trends.

### Descriptive analysis

**Fig. 2:**
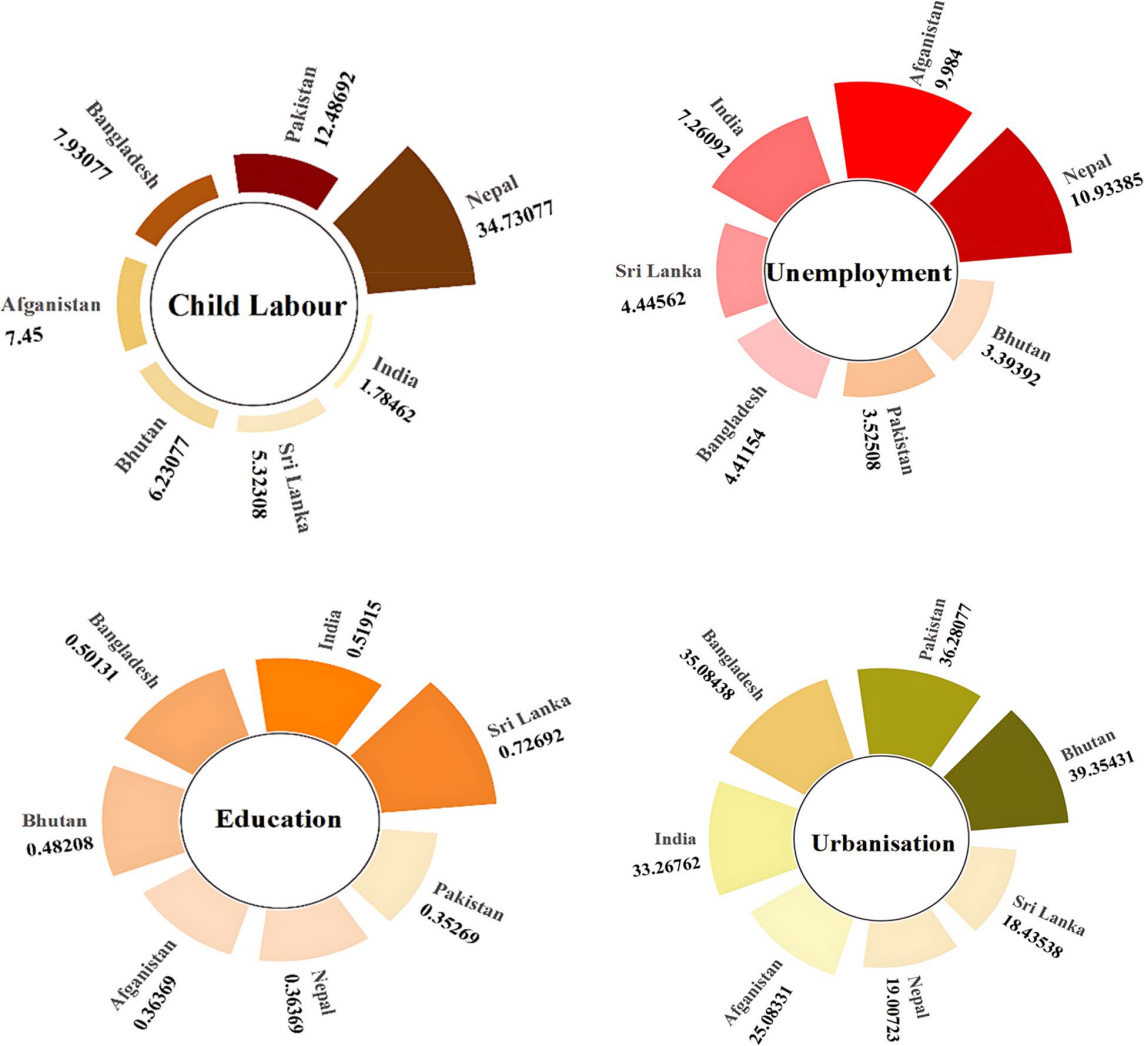

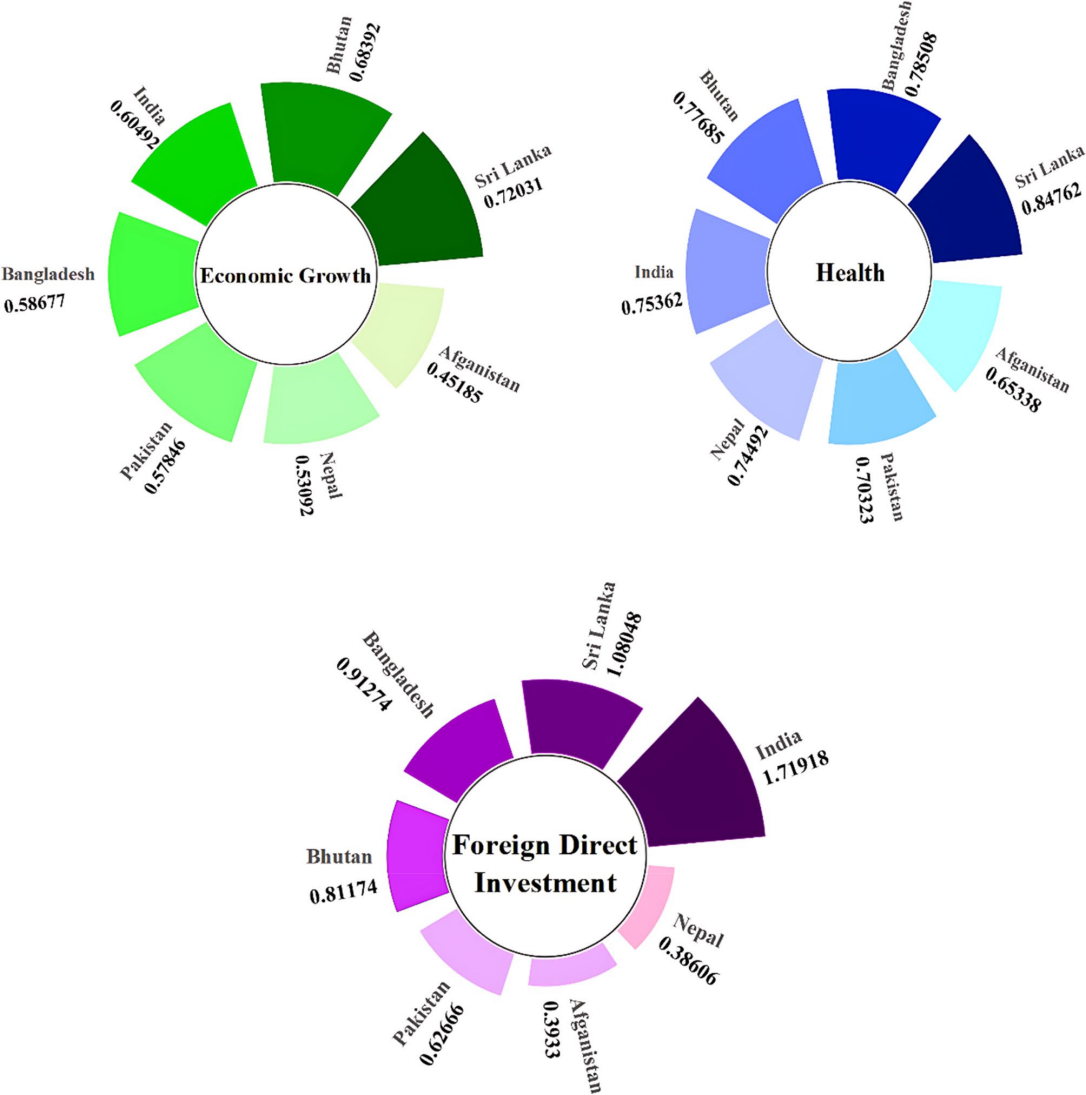
Radial Bar Plots Showing Country-Wise Average Values of Key Socio-Economic Variables Across SAARC Countries. Source: Authors Illustrations based on DOL [9], Global Data Lab [98], Our World in Data [99], World Bank [100]. Note: Fig. 2 presents radial bar plots showing the average values of each key variable for each SAARC country. Each plot represents a different variable, and the length of the bars reflects the country-specific average for that variable. This visualization allows readers to easily compare countries across all indicators, providing context for the cross-country differences observed in child labour determinants

The descriptive statistics, presented in Appendix 2, reveal significant socio-economic disparities across SAARC countries, which are visualised in the radial plots in Fig. 2. These plots highlight clear contrasts in child labour prevalence and its underlying determinants. Nepal stands out with the highest average child labour rate (34.73%), while India records the lowest (1.79%). The shape of the radial plot for Nepal, with low education and health indices coupled with high unemployment, reflects the structural vulnerabilities that sustain high child labour rates. In contrast, India’s stronger performance in education, health, and economic growth is clearly visible in its wider distribution along these axes, explaining its much lower rate of child labour.

Education levels show notable variation across the region, with Sri Lanka displaying the highest mean index (0.727) and Pakistan the lowest (0.353). In Fig. 2, Sri Lanka’s wider education and health bands correspond to its lower incidence of child labour, while Pakistan’s compressed profile highlights the constraints imposed by weak educational access. Health disparities follow a similar pattern, with Sri Lanka leading (0.848) and Afghanistan lagging (0.653). The visual clustering in the plots shows how higher health outcomes are associated with lower reliance on child labour.

Economic growth, unemployment, FDI, and urbanisation also display uneven regional patterns. Sri Lanka records the strongest growth rate (0.72), while Afghanistan has the weakest (0.452), as reflected in their opposing radial shapes. Nepal’s high unemployment (10.93%) is striking in the figure, contrasting with Bhutan’s much lower rate (3.39%). Similarly, India’s leading position in FDI inflows (1.719) is clear, while Nepal’s low level (0.386) reflects its limited industrialisation. Urbanisation adds another layer of complexity: Bhutan, the most urbanised country (39.35%), shows higher exposure to informal urban labour, whereas Sri Lanka, with the lowest urbanisation (18.44%), demonstrates lower child labour rates.

Overall, the radial plots and heatmaps illustrate that countries such as Sri Lanka and India, with stronger education and healthcare systems, higher economic stability, and lower unemployment, occupy more favourable positions across the axes, corresponding with lower child labour rates. In contrast, Nepal, Afghanistan, and Pakistan show constricted profiles, reflecting multiple overlapping disadvantages. These visual patterns underline the importance of considering socio-economic disparities holistically, as the descriptive analysis already hints at the structural factors that the econometric analysis confirms.

**Table 2:** Kao panel cointegration test results for child labour and Socio-Economic determinants

Country	Cointegration unit root					LTR
	MDFt	DFt	ADFt	UMDFt	UDFt	
Afghanistan	−1.6817**	−4.5904***	−2.8289***	−4.2370***	−5.4398***	Yes
Bangladesh	−4.8862***	−5.6626***	−1.2089	−4.8862***	−5.6626***	Yes
Bhutan	0.4944	−0.5832	−0.0008	−3.3866***	−3.6098***	Yes
India	−0.8471	−1.8410**	−1.3383*	−2.1593**	−2.3488***	Yes
Nepal	−3.6577***	−4.0082***	−1.4848*	−3.6577***	−4.0082***	Yes
Pakistan	0.6183	−1.9846**	0.3145	−4.8825***	−8.2810***	Yes
Sri Lanka	−3.4947***	−3.7178***	−2.8574***	−3.4947***	−3.7178***	Yes

Table 2 presents the Kao panel cointegration test results, assessing the long-term equilibrium relationship between child labour and the selected socio-economic variables for SAARC countries. Significant test statistics indicate the presence of stable, long-run associations at the country level. *Significant at 10%, ** significant at 5%, and ***significant 1% significance level. LTR refers to long term relationship

**Table 3:** Pedroni panel cointegration test results for the overall child labour model

Pedroni test for cointegration	
Modified Phillips-Perron t	3.4653***
Phillips-Perron t	−8.7270***
Augmented Dickey-Fuller t	−6.3212***
LTR	Yes

Table 3 presents the Pedroni panel cointegration test results for the overall model, assessing the long-term equilibrium relationship between child labour and the combined socio-economic determinants across SAARC countries. Significant statistics across multiple test dimensions confirm the presence of a robust long-run association at the panel level. *Significant at 10%, ** significant at 5%, and ***significant 1% significance level. LTR refers to long term relationship

### Co-integration analysis

When considered holistically, the Kao cointegration test results given in Table 2 demonstrate that each SAARC country exhibits a stable long-term relationship between child labour and the selected socio-economic factors. These findings confirm that child labour is deeply embedded within broader economic and social structures, rather than being driven by a single variable. In Afghanistan, Bangladesh, India, Nepal, Pakistan, and Sri Lanka, factors such as education, health, economic growth, urbanisation, FDI, and unemployment collectively influence child labour trends, while in Bhutan, where child labour rates are relatively low, urbanisation and employment opportunities still play a role.

To further ensure robustness, the Pedroni cointegration test was conducted for the full SAARC panel and is presented in Table 3. The results showed all three statistics (Modified Phillips–Perron, Phillips–Perron, and Augmented Dickey–Fuller) to be significant, thereby reaffirming the existence of a strong long-term equilibrium relationship between child labour and the socio-economic determinants across the region. Together, the results from both Kao and Pedroni tests strengthen the validity of the findings and highlight that addressing child labour requires a comprehensive, multi-faceted strategy across SAARC nations.

### FMOLS and DOLS regression analysis

To complement the cointegration tests and estimate the long-run effects, the study employs both FMOLS and DOLS which is presented in Table 4. These estimators correct for serial correlation and potential endogeneity in cointegrated systems, thereby allowing reliable long-run parameter estimates.

Results of the FMOLS estimates show that education, health, unemployment, and urbanisation are significant determinants of child labour in the SAARC region. Education and health show strong magnitudes, where higher educational attainment reduces child labour significantly, while lower health outcomes increase child labour. Unemployment and urbanisation also emerge as significant, suggesting labour market structure and demographic pressures play roles in shaping child labour outcomes. Conversely, both economic growth and FDI do not exhibit any significant impact on child labour in FMOLS, which suggests that short-term macroeconomic expansions or foreign investment do not translate directly into reduced child labour. Similarly, the DOLS results show that the overall pattern remains consistent with the FMOLS, which confirms the robustness of the findings. These similarities between FMOLS and DOLS provide reassurance that the results are not estimator-specific but rather reflect stable long-run relationships.

Taken together, the results highlight that structural socio-economic factors such as education, health, labour market dynamics, and urbanisation are more influential in reducing child labour than macroeconomic growth or FDI. The robustness of the findings is further supported by log–log transformed specifications, which are reported in Appendix 4.

**Table 4:** Robustness estimates (FMOLS vs. DOLS)

	FMOLS	DOL
Intercept	−52.4602***	-
EDU	−132.1592*** (10.0285)	−137.1469*** (34.7013)
HEA	167.265*** (26.3287)	257.5486*** (36.4480)
UNE	0.6253** (0.3144)	0.8622* (0.4512)
EG	32.1903 (20.2312)	−10.6672 (33.1167)
UB	−0.7687*** (0.0971)	−0.5572 (0.6846)
FDI	−0.3586 (0.9241)	−1.5478** (0.6859)
R^2^	0.5320	1.3416
Adjusted R^2^	0.5625	−2.5676

Note: Table 4 presents the robustness analysis comparing Fully Modified Ordinary Least Squares (FMOLS) and Dynamic Ordinary Least Squares (DOLS) estimates for the long-term determinants of child labour across SAARC countries. Coefficients are reported with robust standard errors in parentheses. Significance levels are indicated as *10%, **5%, and ***1%. The results show that DOLS estimates are broadly consistent with FMOLS in terms of coefficient signs, magnitudes, and statistical significance, confirming the robustness of the main findings. FMOLS was estimated using cointreg, while DOLS was estimated using xtdolshm with one lag and one lead (nlags(1) nleads(1))

## Discussion

The cointegration results from the Kao and Pedroni tests confirm that child labour maintains a stable long-term relationship with socio-economic factors across SAARC countries. The Kao test identifies this relationship at the country level, while the Pedroni panel results reinforce robustness, with all statistics significant at conventional thresholds. These findings highlight that child labour is structurally linked to economic and social conditions rather than short-term fluctuations. Building on this, the FMOLS and DOLS estimations clarify which factors exert the strongest influence. The close alignment between both estimators further strengthens the reliability of the results.

Education arises as the most significant determinant, displaying a negative coefficient that suggests even moderate improvements in schooling can substantially reduce child labour. This finding supports prior evidence from India and Sri Lanka [10, 101], where universal primary education policies helped to reduce child labour by significant margins [41, 47]. The scale of this difference highlights the persistent education gap in the region and the huge potential returns to further investment in access and quality.

The coefficient for health appears positive, which is a counterintuitive result at first. This however signifies the complex interplay between household health status, income dynamics, and labour decisions. In another context, when the health of children is better, they are more engaged in labour activities because healthier children can contribute more to the household income as shown in studies from Nepal and Bangladesh [31, 35]. In addition, better child health can enable mothers to work more in the labour market and, therefore, better health is indirectly related to more economic activity in families [102]. Nonetheless, while this statistical relationship appears positive, extensive evidence confirms that child labour itself has detrimental health effects, particularly in physically demanding sectors such as manufacturing and construction [32]. Therefore, a positive relationship was observed which is mostly due to the indirect effects of income and measurement processes, and not necessarily to actual welfare increase.

When it comes to unemployment, there is a positive and statistically significant impact on child labour, which shows that adult unemployment encourages dependence on child earnings [103]. This also concurs with the research conducted in India and Pakistan [67, 69], in which the dearth of adult employment drives families to use the child income to cope with the lack of adult employment. The outcome highlights the necessity of inclusive labour market reforms to minimise household vulnerability.

Urbanisation has a weak yet significant negative correlation with the child labour meaning that increased urbanisation is likely to lower child labour. This trend, which is in line with findings in Bhutan, India and Sri Lanka, may be attributed to improved educational access and social awareness in urban areas [37, 60, 61]. However, it is important to note that informal labour markets in cities still pose risks of hidden or unregulated child work.

Economic growth and FDI display statistically insignificant associations with child labour, a finding consistent with prior evidence from Pakistan and regional studies [51, 57]. This suggests that economic expansion alone does not automatically translate into reduced child labour unless accompanied by effective redistribution mechanisms and social protection systems. Structural inequalities within SAARC economies may limit the benefits of growth to certain groups, leaving poorer households dependent on child income.

Cross-country differences further illuminate these structural dynamics. Nepal and Pakistan exhibit relatively stronger persistence of child labour compared to Sri Lanka and Bhutan [13]. Previous studies attribute this to weaker institutional enforcement, limited access to quality education, and higher poverty incidence in Nepal and Pakistan [14, 69]. In contrast, Sri Lanka and Bhutan have achieved greater reductions due to stronger educational attainment, broader healthcare coverage, and policy consistency in child protection initiatives [104]. These contrasts underscore that while the direction of socio-economic effects remains consistent, their magnitude varies according to each country’s institutional strength and social development context.

Overall, findings validate the importance of long-term effect of education and unemployment, the subtle impact of health and urbanisation, and demonstrates that economic growth and investment is not the sole solution to child labour. The similarity of FMOLS and DOLS results supports the strength of such conclusions. To solve the child labour problem in the SAARC countries, thus, relies on concerted efforts that combine education changes, advancement of the labour market and more specific social legislation instead of economic growth.

### Data availability and consistency challenges

A major constraint in analysing child labour across countries, especially countries that does not have a proper data collection system leads to limited availability of harmonised, high-quality data. There is a notable scarcity of the publicly accessible cross country- time series data on child labour. ILO typically reports figures for economically active children aged 5–17, while the World Bank’s World Development Indicators adopt a narrower age bracket of 7–14 years. These definitional differences, combined with irregular survey intervals [105], hinder cross-country comparability and the construction of continuous time-series datasets.

Inconsistencies in survey instruments further complicate interpretation. For example, child labour in India took a sharp increase in 2006 then decreased back to previous levels in 2010 which corresponds to a change in data source from the National Sample Survey to the Demographic and Health Survey [106]. Such artefacts underscore the challenges of merging data across heterogeneous instruments and have led many academic studies to rely on a single survey source, such as UNICEF’s Multiple Indicator Cluster Surveys [105], to maintain internal consistency.

Given these limitations, the present study employs estimate from DOL, which consolidates national statistics with inputs from recognised international agencies including the ILO and UNICEF. Although normal forecasting function such as linear interpolation, was used to address smaller data gaps and construct a balanced panel, this approach prioritised methodological consistency and minimised the distortions that often arise from switching between incompatible data sources. Forecasting helps to mitigate these issues arising from intermittent or sporadic child labour, which is often underreported in household surveys [107]. While this method does not fully address all underlying data limitations, it enables meaningful cross-country comparisons within the SAARC region.

There are several paths for future research expansion regarding the dynamics of child labour, particularly considering the data limitations from sources such as the DOL, UNICEF, and ILO. One significant issue is the frequent lack of updated data, with figures often remaining unchanged over multiple years due to delays in new data availability. This limitation can hinder the accurate depiction of recent trends and shifts in child labour dynamics. Nonetheless, existing datasets still provide invaluable insights into long-term patterns and policy impacts. Similar limitations have been acknowledged in various studies that successfully used historical data from DOL to draw meaningful conclusions about child labour and its determinants [80, 108, 109]. These constraints highlight the importance of continued efforts to improve data collection while also validating the relevance of existing sources for conducting rigorous long-term analyses.

## Conclusion and policy implications

### Summary of key findings

This study examines the long-term relationship between child labour and six socio-economic factors, focusing on the SAARC countries using panel cointegration analysis. Findings confirm that while education and health improvements reduce child labour, economic growth is crucial, particularly in Pakistan and India. However, the effects of urbanisation and unemployment differ. Bhutan and India highlight urbanisation-driven changes in child labour, while unemployment remains a key driver in Pakistan and Sri Lanka. FDI’s impact is complex, highlighting the need for broader welfare policies. Country-wise analysis shows that key influences on child labour differ, reinforcing the need for tailored policy responses.

### Policy implications

To effectively combat child labour, SAARC nations must implement a comprehensive policy framework that addresses its root causes. Strengthening educational policies, expanding access to healthcare, fostering inclusive economic growth, and enforcing labour regulations are crucial steps toward ensuring that economic progress does not come at the expense of children’s rights and well-being.

#### Strengthening education policies

Universal access to quality education is essential in preventing child labour. Governments should enforce compulsory education laws and eliminate financial barriers through tuition subsidies, free school materials, and transport assistance. Many low-income families contend with education costs, resulting in dropouts and child labour. Vocational training can equip older children with employable skills, providing alternatives to labour-intensive jobs. Expanding conditional cash transfer programmes offering financial aid for school attendance can further diminish child labour across SAARC countries.

#### Healthcare interventions

Improving public health can indirectly reduce child labour by relieving the financial pressure caused by illness within families. Expanding child and maternal healthcare, particularly in rural and low-income areas, is critical. Free or subsidised healthcare and preventive services such as immunisation and nutrition programmes can lower household medical costs, allowing families to prioritise education. School-based nutrition initiatives and awareness campaigns can further promote children’s attendance and overall well-being.

#### Inclusive economic and employment policies

While economic growth is mostly taken as a pathway to directly reduce child labour, the results highlight that growth alone is insufficient unless it is inclusive. Policymakers should support small and medium enterprises (SMEs) which create stable adult employment, particularly in rural and informal sectors where child labour is most prevalent. Strengthening labour regulations, enforcing minimum wage standards, and improving working conditions could often ensure that growth benefits vulnerable households. Expanding social protection programmes such as unemployment benefits, food subsidies, and cash transfers can provide essential safety nets, preventing families from depending on children’s income during periods of financial stress.

#### Ethical and responsible FDI policies

FDI has a significant impact on the labour market, and ethical investment policies are necessary to prevent the exploitation of child labour. Governments ought to implement stringent corporate social responsibility (CSR) legislation to ensure that foreign multinationals comply with both national and international labour standards. Encouraging investments in skilled labour sectors, such as manufacturing, services, and technology, will foster sustainable employment for adults and reduce dependence on child labour. Furthermore, rigorous monitoring and reporting systems must be established to uphold transparency and accountability in foreign business practices to prevent exploitative labour practices.

#### Urban planning and child labour regulations

As urbanisation accelerates, addressing child labour in informal sectors demands comprehensive planning. Migrant families frequently encounter inadequate infrastructure and housing, compelling children into exploitative employment. Governments should enhance urban infrastructure to ensure affordable housing, healthcare, and education access. The redevelopment of slums, incorporating integrated social services, can improve living conditions and diminish the risks of child labour. Stricter labour regulations and incentives for businesses to prioritise adult workers over children can further reduce urban child labour.

#### Regional collaborations within SAARC

As child labour is a cross-border issue, regional collaboration among SAARC countries is essential for effective intervention. The SAARC countries must share best practices and effective policy models to combat child labour. Regional agreements on child labour legislation can promote a uniform legal framework that eliminates regulatory loopholes and inconsistencies across borders. Furthermore, cross-border initiatives should be designed to address trafficking, forced labour, and migration-related child labour exploitation. Implementing joint monitoring systems and collaborative enforcement measures can maximise the efficiency of national efforts while fostering a regional consensus to eradicate child labour.

While the proposed policy interventions are designed to reduce child labour effectively, their implementation may face practical challenges. Resource constraints, such as limited funding or insufficient trained personnel, could slow the rollout of education and health programs. Political and bureaucratic barriers, including policy inertia or competing priorities, may also affect timely execution. Additionally, socio-cultural factors, such as local norms and household perceptions of child labour, could influence the uptake of interventions. Recognizing these challenges underscores the importance of gradual, context-sensitive implementation strategies and targeted support to ensure that policy measures achieve their intended impact.

### Potential limitations of the study

One key limitation of this study is the scarcity of past research on child labour in SAARC countries, making it challenging to build upon existing literature. Furthermore, child labour data for the region, particularly before 2010, is limited and inconsistent, which may affect the depth of historical analysis. Child labour is also often underreported, especially in informal, unpaid, or domestic work, and children outside household structures may be excluded from survey data. These issues reduce the reliability and completeness of available data and should be considered when interpreting the findings.

Another limitation relates to data forecasting. In cases where gaps existed, linear interpolation was applied to maintain consistency in the panel dataset. While widely used for its simplicity, interpolation assumes smooth changes over time and may not capture sudden shocks or structural breaks. This introduces some uncertainty into the estimates, which should be kept in mind when interpreting the results. Future studies could improve robustness by employing more sophisticated forecasting techniques. A further challenge concerns cross-country comparability. Child labour statistics in the SAARC region are drawn from national surveys and international databases that vary in scope, coverage, and definitions. Although harmonised datasets were used where possible, underlying disparities in reporting standards and collection methods may introduce bias into cross-country comparisons.

The study’s use of proxy variables also imposes constraints. For example, adult unemployment and health indicators were included to represent socio-economic conditions, but these proxies may not fully reflect the immediate household-level drivers of child labour. Decisions around child labour often respond to short-term shocks such as sudden income loss, natural disasters, or illness, which are not adequately captured by aggregate annual data. Similarly, the Education Index reflects long-term educational development but does not directly capture school attendance or dropout rates, which are more closely linked to child labour.

Finally, the methodology focuses on long-run relationships by applying cointegration techniques such as FMOLS and DOLS. While this approach is appropriate for uncovering structural determinants, it does not account for short-run transitional dynamics. Temporary rises in unemployment, economic crises, or policy interventions may influence child labour in the short term but are not fully captured within the current framework. Furthermore, potential short-run feedback effects between socio-economic conditions and child labour, such as those captured through panel-VECM or Granger causality frameworks, are acknowledged but remain beyond the scope of the present study.

### Future research directions

While this study examines the long-term relationship between socio-economic factors and child labour in SAARC countries, further research remains necessary. Qualitative methods may provide deeper insights into experiences of child labour and the effectiveness of policies. Investigating short-term economic shocks, such as pandemics, would also be beneficial. Sector-specific analyses would elucidate how international investments impact child labour. While FMOLS/DOLS mitigate reverse causality, the issue cannot be fully excluded; future work could apply panel-VECM approaches to capture short-run dynamics. Moreover, future research should include gender-based perspectives for a more nuanced understanding. Additionally, exploring other critical variables, such as cultural factors, social norms, access to social protection, and the role of governance, would enhance understanding and contribute to more targeted strategies for combating child labour.

## Supplementary Information

Supplementary Material 1: Appendix 1. Data File

Supplementary Material 2: Appendix 2. Descriptive Statistics. 

Supplementary Material 3: Appendix 3. Preliminary Test Results

Supplementary Material 4: Appendix 4. Log-Log Robustness Estimates (FMOLS vs. DOLS). 

## Data Availability

All data generated or analysed during this study are included in this published article and its supplementary information files.
