# Supplementary material for: Breaking the cycle: long-term socio economic determinants of child labour in SAARC countries
Source: BMC Public Health. 2025 Nov 19;25:4040. doi: 10.1186/s12889-025-25399-w (PMC12628829; doi:10.1186/s12889-025-25399-w)
Supplement: Supplementary file 2 — Supplementary Material 2: Appendix 2. Descriptive Statistics. [file 12889_2025_25399_MOESM2_ESM.docx]

**Appendix 2: Descriptive Statistics
Afghanistan**

|  | N | MEAN | SD | MIN | MAX |
| --- | --- | --- | --- | --- | --- |
| Child Labour | 13 | 7.45 | 2.330 | 4.3 | 12 |
| Education | 13 | .364 | 0.025 | .318 | .397 |
| Health | 13 | .653 | 0.012 | .628 | .67 |
| Economic Growth | 13 | .452 | 0.023 | .391 | .47 |
| Unemployment | 13 | 9.984 | 2.083 | 7.753 | 14.1 |
| FDI | 13 | .393 | 0.336 | .065 | 1.203 |
| Urbanisation | 13 | 25.083 | 0.921 | 23.737 | 26.616 |

**Bangladesh**

| Child Labour | 13 | 7.931 | 3.185 | 4.3 | 13.6 |
| --- | --- | --- | --- | --- | --- |
| Education | 13 | .501 | 0.057 | .426 | .578 |
| Health | 13 | .785 | 0.025 | .748 | .826 |
| Economic Growth | 13 | .587 | 0.028 | .544 | .631 |
| Unemployment | 13 | 4.412 | 0.545 | 3.379 | 5.436 |
| FDI | 13 | .913 | 0.451 | .355 | 1.735 |
| Urbanisation | 13 | 35.084 | 3.007 | 30.462 | 39.711 |

**Bhutan**

| Child Labour | 13 | 6.231 | 5.933 | 3.8 | 19.6 |
| --- | --- | --- | --- | --- | --- |
| Education | 13 | .482 | 0.056 | .407 | .557 |
| Health | 13 | .777 | 0.018 | .745 | .804 |
| Economic Growth | 13 | .684 | 0.018 | .652 | .706 |
| Unemployment | 13 | 3.394 | 1.149 | 2.05 | 5.947 |
| FDI | 13 | .812 | 1.237 | -.639 | 4.405 |
| Urbanisation | 13 | 39.354 | 2.891 | 34.793 | 43.686 |

**India**

| Child Labour | 13 | 1.785 | 0.708 | 1.4 | 3.3 |
| --- | --- | --- | --- | --- | --- |
| Education | 13 | .519 | 0.029 | .461 | .569 |
| Health | 13 | .754 | 0.022 | .722 | .783 |
| Economic Growth | 13 | .605 | 0.027 | .563 | .641 |
| Unemployment | 13 | 7.261 | 0.866 | 4.822 | 7.859 |
| FDI | 13 | 1.719 | 0.313 | 1.313 | 2.406 |
| Urbanisation | 13 | 33.268 | 1.605 | 30.93 | 35.872 |

**Nepal**

| Child Labour | 13 | 34.731 | 4.590 | 20.4 | 37.2 |
| --- | --- | --- | --- | --- | --- |
| Education | 13 | .364 | 0.025 | .318 | .397 |
| Health | 13 | .745 | 0.016 | .72 | .777 |
| Economic Growth | 13 | .531 | 0.021 | .498 | .558 |
| Unemployment | 13 | 10.934 | 0.756 | 10.389 | 12.975 |
| FDI | 13 | .386 | 0.169 | .134 | .677 |
| Urbanisation | 13 | 19.007 | 1.520 | 16.768 | 21.451 |

**Pakistan**

| Child Labour | 13 | 12.487 | 2.186 | 9.8 | 15.9 |
| --- | --- | --- | --- | --- | --- |
| Education | 13 | .353 | 0.018 | .321 | .369 |
| Health | 13 | .703 | 0.012 | .684 | .719 |
| Economic Growth | 13 | .578 | 0.015 | .557 | .602 |
| Unemployment | 13 | 3.525 | 1.851 | .653 | 6.338 |
| FDI | 13 | .627 | 0.182 | .343 | 1.028 |
| Urbanisation | 13 | 36.281 | 0.871 | 34.997 | 37.731 |

**Sri Lanka**

| Child Labour | 13 | 5.323 | 4.359 | .8 | 9.2 |
| --- | --- | --- | --- | --- | --- |
| Education | 13 | .727 | 0.015 | .71 | .754 |
| Health | 13 | .848 | 0.018 | .819 | .871 |
| Economic Growth | 13 | .72 | 0.018 | .684 | .74 |
| Unemployment | 13 | 4.446 | 0.419 | 3.88 | 5.364 |
| FDI | 13 | 1.08 | 0.347 | .515 | 1.708 |
| Urbanisation | 13 | 18.435 | 0.279 | 18.196 | 19.026 |
