# Supplementary material for: Breaking the cycle: long-term socio economic determinants of child labour in SAARC countries
Source: BMC Public Health. 2025 Nov 19;25:4040. doi: 10.1186/s12889-025-25399-w (PMC12628829; doi:10.1186/s12889-025-25399-w)
Supplement: Supplementary file 3 — Supplementary Material 3: Appendix 3. Preliminary Test Results [file 12889_2025_25399_MOESM3_ESM.docx]

**Appendix 3: Preliminary Test Results**

| **Country** | **Child Labour** |
| --- | --- |
| Child labour | –0.787 |
| Education | –1.597 |
| Health | –1.778 |
| Economic growth | –1.066 |
| Unemployment | –2.318* |
| FDI | –1.607 |
| Urbanisation | +1.306 |

**Appendix 3A: Testing for unit roots [Pesaran CADF (CIPS)]**

Note: Ho denotes Panels contain unit roots, and Ha is Panels are stationary, ***significant at 1% significance level

| **Test** | **Statistic** |
| --- | --- |
| Pesaran CD test (2004) | –1.769* |

**Appendix 3B: Cross-sectional dependence (Pesaran CD test)**

Note: *Significant at 10%, ** significant at 5%, and ***significant 1% significance level.

| **Test** | **Statistic** |
| --- | --- |
| Pesaran & Yamagata (2008) Δ | 2.439** |
| Pesaran & Yamagata (2008) Δ_adj | 3.933*** |

**Appendix 3C: Slope heterogeneity (Pesaran & Yamagata)**

Note: *Significant at 10%, ** significant at 5%, and ***significant 1% significance level.

**Appendix 3D: VIF Test Results for Multicollinearity**

| **Variable** | **VIF** | **1/VIF** |
| --- | --- | --- |
| Economic Growth | 8.53 | 0.1172 |
| Health | 6.82 | 0.1466 |
| Education | 4.63 | 0.2159 |
| Unemployment | 2.74 | 0.3651 |
| Urbanisation | 1.73 | 0.5764 |
| FDI | 1.11 | 0.8995 |
| **Mean VIF** | 4.26 | — |
