# Supplementary material for: Breaking the cycle: long-term socio economic determinants of child labour in SAARC countries
Source: BMC Public Health. 2025 Nov 19;25:4040. doi: 10.1186/s12889-025-25399-w (PMC12628829; doi:10.1186/s12889-025-25399-w)
Supplement: Supplementary file 4 — Supplementary Material 4: Appendix 4. Log-Log Robustness Estimates (FMOLS vs. DOLS). [file 12889_2025_25399_MOESM4_ESM.docx]

**Appendix 4: Log-Log Robustness Estimates (FMOLS vs DOLS)**

| **Variables** | **FMOLS (ln_childlabour)** | **DOLS (ln_childlabour)** |
| --- | --- | --- |
| ln_economicgrowth | -2.051**  (0.730) | -5.185  (3.163) |
| ln_health | 9.880***  (1.334) | 18.066***  (6.158) |
| ln_education | -4.544***  (0.311) | -5.255  (3.465) |
| ln_unemployment | -0.273**  (0.097) | -0.199  (0.338) |
| ln_fdi | -0.332***  (0.115) | -0.578  (0.361) |
| ln_urbanisation | -1.043***  (0.158) | -1.532  (4.263) |
| Constant | 4.201***  (0.585) | — |
| **R-squared** | 0.548 | 0.450 |

Notes: Robust standard errors in parentheses. *** p<0.01, ** p<0.05, * p<0.1. FMOLS estimated using cointreg; DOLS estimated using xtdolshm with nlags(1) nleads(1).
